# Supplementary material for: Domain generalization enables general cancer cell annotation in single-cell and spatial transcriptomics
Source: Nat Commun. 2024 Mar 2;15:1929. doi: 10.1038/s41467-024-46413-6 (PMC10908802; doi:10.1038/s41467-024-46413-6)
Supplement: Supplementary file 1 — Supplementary Information [file 41467_2024_46413_MOESM1_ESM.pdf]

Supplementary Information for

Domain generalization enables general cancer cell annotation in single-cell and spatial transcriptomics

Zhixing Zhong[#1,2], Junchen Hou[#3], Zhixian Yao[#2], Lei Dong[#4], Feng Liu[5], Junqiu Yue[6], Tiantian Wu[3], Junhua Zheng[2], Gaoliang Ouyang[3], Chaoyong Yang[1,2,7], Jia Song*[2]

[1]Institute of Artificial Intelligence, Department of Chemical Biology, College of Chemistry and Chemical Engineering, Xiamen University, Xiamen 361102, China.

[2]Institute of Molecular Medicine, Department of Urology, Renji Hospital, School of Medicine, Shanghai Jiao Tong University, Shanghai 200127, China.

[3]School of Pharmaceutical Sciences, State Key Laboratory of Cellular Stress Biology, School of Life Sciences, Xiamen University, Xiamen 361102, China.

[4]Department of Pathology, Shanghai Jiao Tong University Medical School Affiliated Ruijin Hospital, Shanghai 200025, China.

[5]School of Computing and Information Systems, The University of Melbourne, Carlton, Melbourne, VIC 3053, Australia.

[6]Department of Pathology, Hubei Cancer Hospital, Tongji Medical College, Huazhong University of Science and Technology, Wuhan 430030, China.

[7]Innovation Laboratory for Sciences and Technologies of Energy Materials of Fujian Province (IKKEM), Xiamen 361005, China.

Corresponding authors: songjiajia2010@shsmu.edu.cn (Jia Song)

These authors contributed equally: Zhixing Zhong, Junchen Hou, Zhixian Yao, Lei Dong

6 Supplementary Notes
17 Supplementary Tables
16 Supplementary Figures

Supplementary Notes

Supplementary Note 1: Details of the interpretability module of Cancer-Finder

Saliency Map[1] was utilized to determine the model's interpretability. Specifically, the gradient of the loss function can be obtained as follow based on backpropagation:

$$\mathbf{W} = \{w_1, w_2, \dots, w_m\}^T = \text{Gradient}\big(\text{Loss}(\boldsymbol{\theta})\big) \quad (1)$$

where Loss($\boldsymbol{\theta}$) is the loss function, W is the gradient containing m elements, and m is the number of features. m = 5000 for ST and m = 4572 for single cell data. After one training loop, the salience value for each feature (gene) was defined as follows:

$$\text{salience}_{\text{Gene } i} = \text{sum}(w_{\text{Gene } i}) = \sum_{e=1}^{n} |w_i^e| \quad (2)$$

where n is the number of samples within a training loop. The salience value indicates the contribution of each gene to the training. The genes were subsequently ranked based on their contributions during training. After the top ten genes remained unchanged for 20 epochs, it was concluded that the ranking of the genes, in terms of their contribution to the training, had reached a stable state. In our experiments, this occurred between the 69-89th training rounds, at which point we selected the ten most significant genes for co-localization and subsequent analysis.

Supplementary Note 2: Details of Data Preprocessing

Dataset collection and merging. As training sets, matrices of single-cell expression obtained from TISCH[2], which contain a total of 79 single-cell datasets from the tumor microenvironment, were utilized. Following the removal of 5 mouse datasets, 74 human datasets remained. We annotate each cell in these datasets according to the 'Celltype' column in the metadata file provided by the database. This column includes the five label categories: Malignant, Immune, Stromal, HSC, and Others. Stromal and immune cells were utilized as non-malignant cells. Due to the difficulty of determining the malignancy status of cells annotated as HSC and Others and the limited amount of data in these two categories, we eliminated 68,759 HSC and Others cells, leaving 73 data sets containing 1,925,117 cells, including 378,691 malignant cells and 1,546,426 non-malignant cells.

Subsequently, we merged 73 datasets into 17 tissue datasets including bladder, blood, bone, brain, breast, colorectal, eye, head and neck, kidney, liver, lung, lymph node, nervous system, pancreas, pelvis, skin, and stomach based on the tissue information provided by the database. Due to the absence of malignant cells, 3 of the tissue data (bladder, kidney, lymph node, a total of 6 datasets containing 162,846 cells) were discarded, leaving 14 tissue data (67 datasets containing 1,762,271 cells) for further analysis.

In the context of spatial transcriptomic data, we utilized the same treatment protocol and obtained 14 sections of 10x Visium ST data. In addition to the 10x Genomics website (https://www.10xgenomics.com/resources/, including 2 BRCA and 1 OV sections), these data were collected from three independent studies (including 3

CRC[3], 4 HCC[4], 1 ICC[4], and 3 RCC[5] slides). The collected data were then classified into six distinct tissue categories, namely mammary, colorectal, renal, hepatic, biliary, and ovarian. The classification of non-malignant and malignant spots was referenced from a previous study[6] and revised by pathologists, and some slides without available annotation were manually annotated by pathologists directly. A HCC section (HCC-4L) was used to determine the final training round, while the remaining datasets were used for training.

Balanced sampling.To achieve a 1:1 ratio between malignant and non-malignant cells in each tissue dataset, malignant and non-malignant cells were down-sampled. In four tissues (bone, brain, nerve, and eye), there are more malignant cells. Thus, all non-malignant cells were utilized, while malignant cells were randomly down-sampled to match the number of non-malignant cells. In the remaining ten tissues, non-malignant cells were down-sampled and all malignant cells were utilized. Lastly, the 1:1 balanced dataset consists of 340,178 cells from 14 tissues (named as TICSH data 1) .

These training data have already been normalized by the developer of the TICSH database (the 'NormalizeData' function from Seurat was used for global scaling normalization), and have thus been used directly for subsequent analysis. For external validation sets and test sets, the raw count is normalized using the same method. ST data is handled in the same manner.

Feature selection. Based on TICSH data 1, two sets of features were extracted and were compared for model training. The first set, feature concatenation, contained 28256 genes expressed in at least one tissue. The second set, the feature intersection set, contained 4572 genes expressed in all 14 tissues. The performance of these two feature sets was evaluated by randomly selecting 4/5 of the training data as the training set and 1/5 as the internal validation set. The model trained on the concatenation set had lower accuracy (77.34%) than the intersection set (92.75%), so genes in the intersection set were used as selected features in subsequent analysis. The training set of ST data had a large intersection of features (31908 genes). Training on such a large number of features is excessively time-consuming, so we chose the top 5000 genes with the highest cellular expression variance as selected features.

Model parameter determination. β serves as an important hyperparameter in the risk extrapolation method, controlling the balance between reducing the average risk and enforcing equality of risks, with beta → 0 recovering ERM, and beta → ∞ leading to focus entirely on making the risk equal[7]. Using 5-fold cross-validation, several β values were accessed. β was set to 0, 0.2, 0.4, 0.6......1.8, 2 and 4, 6, 8 for model training. Here, models were trained and evaluated using the scRNA-seq training set with 340,178 cells. Models training was terminated when the accuracy of breast cancer data prediction reached a stable maximum. As shown in **Supplementary Figure 15**, the most effective β fluctuates around 1 (0.6-2) during 5-fold cross-validation. Considering that the larger the β, the less weight is given to the evaluation of the total training risk in the loss function, β = 1 was chosen to control the overall training risk (cross-entropy loss).

Cross-validation. For a thorough assessment of the model, leave-datasets-out, leave-cells-out, and leave-one-cancer-type-out cross-validations were carried out. Leave-

datasets-out cross-validation was performed by excluding scRNA-seq data from one dataset, training Cancer-Finder with data from other datasets, and predicting cell annotation labels for the excluded dataset. Leave-one-cancer-out cross-validation was performed by excluding scRNA-seq data from one tissue, training Cancer-Finder with data from other tissues, and predicting cell annotation labels for the excluded tissue. In this study, leave-cells-out is commonly employed for 5-fold cross-validation by randomly selecting 80% of cells for model training and 20% for model validation.

In the single-cell analysis, TISCH data 1 was used to evaluate the model. The STAD (Stomach) dataset (GSE134520) has the lowest accuracy in the result of leave-one-cancer-type-out cross-validation. By comparing the database (TISCH) annotation to the original study[8], annotation errors in this dataset were identified (according to the annotations of the TISCH database, malignant cells are present in the samples of patients with NAG, CAG, and IM diseases, but not in the samples of patients with early gastric cancer (EGC)). Thus, this dataset was removed. In the result of leave-datasets-out cross-validation, three datasets (NSCLC_GSE131907, BRCA_GSE138536, and Glioma_GSE138794) show low accuracies, and there are conflicts between the original studies' annotation and TISCH data. These datasets were deleted as well. After balanced sampling, 63 datasets from 13 tissues consisting of 328,230 cells (defined as TISCH data 2) were used for subsequent model training.

Model training for external validation. Model training for external validation involved training Cancer-Finder five times to complete the five repetitions, without any fine-tuning applied to the test data. Specifically, the training dataset from TISCH (328,230 cells) was divided into five folds. In each time, the model underwent training using four out of the five folds and was evaluated on the external validation sets.

The decision to conduct multiple training sessions stems from two primary reasons. Firstly, since most of the other four algorithms exhibit some level of randomness in their results across runs, we ran them five times to better reflect the randomness and accuracies of these methods. Secondly, even though Cancer-Finder consistently produces uniform predictions on the external datasets, its training process and training data introduce a degree of randomness that may result in fluctuations. Therefore, to comprehensively showcase Cancer-Finder's accuracies, we performed five training sessions to capture the potential range of its performance. For ikarus (retrained), we employed the same strategy.

Supplementary Note 3: Details on evaluation metrics

We use two distinct terms for the same metric because the label reliability in the benchmark datasets differs. We use accuracy to describe the accuracy of Cancer-Finder on the gold standard dataset because the reference labels of cells on the gold standard dataset are highly trustworthy. Because the reference labels on the silver standard dataset were annotated by other studies and may not be completely reliable, we use

similarity to characterize Cancer-Finder's prediction of labels on the silver standard dataset.

Supplementary Note 4: Specifics of the calculation time evaluation

The calculation time of each algorithm was assessed using scRNA-seq data from mixed cell lines (dataset 1). To accurately measure the execution time of the algorithms on datasets with varying sizes, samples containing 100, 1000, 10,000, 100,000 and 1,000,000 cells were created. There are 5001 cells in the original scRNA-seq data for mixed cell lines. We randomly downsampled the data in order to obtain the 100-cell and 1,000-cell datasets. For other datasets, random upsampling was employed. The time evaluated encompassed the interval between data loading and result generation. We used the 'time' package to record the time for Cancer-Finder and Ikarus[9], which were executed using Python commands. The time of CopyKAT[10] was derived from its log file. Linux's 'date' command was utilized to record the time for CaSee[11], which was executed via a shell command. For SCEVAN[12], we used the R function 'Sys.time()' to record the time, which is run with the R command. To test the speed of Cancer-Finder further, we stored the expression matrix in binary files using the Python "pickle" package, taking 4.15 seconds and 39.46 seconds for the inference of 10,000 and 100,000 cells, respectively.

Supplementary Note 5: Applications to spatial transcriptomic (ST) data derived from diverse spatial transcriptomic sequencing platforms

Other than the commercial platform 10X Visium, which has been utilized in a variety of applications, other platforms have fewer use cases, and even fewer data on cancer tissues. In this situation, it is challenging to collect a large enough training set (including at least 2-3 types of cancer data) to train a pre-trained model on data from multiple platforms. Consequently, this study focuses primarily on predicting data from other platforms using the training results from the existing training set (the pre-trained models based on scRNA-seq data and 10X Visium data). Here, we primarily focus on making predictions using datasets from one imaging-based technique (MERFISH[13]) and two sequencing-based techniques with different resolutions, namely Slide-seq[14] and legacy ST[15]. Detailly, four MERFISH slides (https://info.vizgen.com/ffpe-showcase), four Slide-seq slides[16] and two legacy ST slides[17] were downloaded.

Considering that MERFISH data are most similar to the single-cell form, we initially trained the model with scRNA-seq data. Here, we trained the model using the single-cell sub-matrix (containing 550 genes measured in the MERFISH data), and utilized the training results to predict malignant cells in the MERFISH dataset. As shown in **Supplementary Figure 7a** and **Figure 4**, in the case of using a suitable Softmax threshold, Cancer-Finder has a high degree of accuracy on the MERFISH data. Notably, we observed that Cancer-Finder may generate false positives when the pre-trained model was applied directly to MERFISH data with the default softmax threshold (threshold = 0.5) because single-cell data and MERFISH data are not identical. Based on a MERFISH slide, the ROC curve was used to determine the optimal threshold

(threshold = 0.9766), Cancer-Finder was able to accurately predict MERFISH data (accuracy: 70.69–83.84 %, AUC: 0.7707–0.8969).

Similarly, we have expanded our predictions to Slide-seq data. This is a second-generation sequencing-based ST technology with near single-cell resolution (spot diameter of 10um), so we still made predictions with the pre-trained model we obtained on scRNA-seq dataset, and the results demonstrated that Cancer-Finder performs exceptionally well on the majority of the datasets (**Supplementary Figure 7b**).

Lastly, we attempted to extend the model to legacy ST slides with a larger spot (spot diameter of 100um) and made predictions utilizing a pre-trained model trained on 10x Visium slides. As shown in **Supplementary Figure 7c**, the performance of Cancer-Finder varies across datasets (slide 1: accuracy=0.8050, AUC=0.8227; slide 2: accuracy=0.5765, AUC=0.5650).

Supplementary Note 6: The rationale for choosing V-REx

In cell classification and annotation, neural networks have numerous applications and perform exceptionally well[18]. Tumor heterogeneity creates genetical differences in the distribution of gene expression in different cancers[19], whereas neural networks are sensitive to distribution shift[20]. Domain generalization is specifically designed for this type of problem[21]. Among domain generalization strategies, V-REx[7] (risk exploration) has a simple and efficient mathematical form, which makes its computation less complex and computationally burdensome, and therefore more suitable for training on large datasets. In addition, nine domain generalization strategies were evaluated by Wang et al.[21], and the evaluated results are available at github (https://github.com/jindongwang/transferlearning/tree/master/code/DeepDG) and detailed in **Supplementary Table 17**.

Based on the results, V-REx exhibits consistent and robust performance across four sets of evaluations on two datasets (PACS dataset[22] and Home-Office dataset[23]), consistently placing in the top three in three of these evaluations. Overall, we are confident that this approach can significantly enhance the annotation of the malignant state within the tumor microenvironment across various types of cancer.

Supplementary Tables

Supplementary Table 1. Introduction of external test datasets used in this study

Data Sets	Cancer Type	Tissue	No. of cancer Cells	Malignant Cell percent (%)	Sequencing Platform	Dataset type
10k Peripheral Blood Mononuclear Cells[24] (dataset 1**)**	None	PBMC	0	0%	10x Genomics	Gold standard
Tian, L. *et al* [25] (dataset 2)	Lung adenocarcinoma (LUAD)	Lung (5 Cell lines)	5001	100%	10x Genomics	Gold standard
Riemondy, K. A. *et al* [26] (dataset 3)	Medulloblastom a (MB)	Brain	34243	85.72%	10x Genomics	Silver standard
Bondoc, A. *et al*[27] (dataset 4)	Hepatoblastoma (HB)	Liver	52431	78.13%	10x Genomics	Silver standard
Szczerba, B. M. *et al*[28], Donato, C. *et al*[29] (dataset 5)	Breast Cancer (BRCA)	CTC (Breast)	276	77.31%	Smart-seq2	Silver standard
Qian, J. *et al* [30] (dataset 6)	Breast Cancer (BRCA)	Breast	16235	36.88%	10x Genomics	Silver standard
Qian, J. *et al* [30] (dataset 7)	Ovarian Cancer (OV)	Ovary	14134	31.33%	10x Genomics	Silver standard
Qian, J. *et al* [30] (dataset 8)	Color and Rectal Cancer (COADREAD)	Colon and Rectum	11103	24.85%	10x Genomics	Silver standard
Qian, J. *et al* [30] (dataset 9)	Lung Cancer (LUNG)	Lung	12312	13.16%	10x Genomics	Silver standard
Eberhardt, C. S. *et al*[31] (dataset 10)	Head and Neck Cancer (Head&Neck)	Head and Neck	0	0%	10x Genomics	Silver standard

Supplementary Table 2. Details of annotation strategies of external test datasets used in their original studies

Data Sets	Annotation Methods	Description
10k Peripheral Blood Mononuclear Cells [24] (dataset 1)	Pure normal cells	Peripheral blood mononuclear cells from a healthy donor
Tian, L. *et al* [25] (dataset 2)	Pure cancer cells	Mixed human lung adenocarcinoma cell lines including H2228, H1975, A549, H838 and HCC827
Riemondy, K. A. *et al* [26] (dataset 3)	Clustering combined with inferCNV copy number variation inference for manual annotation	scRNA-seq of human medulloblastoma samples
Bondoc, A. *et al*[27] (dataset 4)	Clustering followed by marker gene annotation	scRNA-seq of human hepatoblastoma samples
Szczerba, B. M. *et al*[28], Donato, C. *et al* [29] (dataset 5)	Experimental enrichment of specific cells	Malignant circulating tumor cells (CTCs) and non-malignant cells obtained from blood samples
Qian, J. *et al*[30] (dataset 6-9)	Clustering followed by marker gene annotation	A pan-cancer study, including scRNA-seq of ovarian cancer, color&rectal cancer, lung cancer and breast samples
Eberhardt, C. S. *et al*[31] (dataset 10)	Pure normal cells (T cells)	Enrichment of T cells using experiments

Supplementary Table 3. Introductions of algorithms compared in this study

Algorithms	URL	Training dataset	Description
CopyKAT[10]	https://www.nature.com/articles/s41587-020-00795-2	Unnecessary	Copy number variation inference
Casee[11]	https://www.nature.com/articles/s41388-022-02478-5	Bulk sequence data	Transfer learning using bulk data as the source domain
Ikarus[9]	https://doi.org/10.1186/s13059-022-02683-1	Single-cell sequence data, with performance varying with the choice of training set	Logistic regression
SCEVAN[12]	https://www.nature.com/articles/s41467-023-36790-9	Unnecessary	Detecting the clonal copy number substructure

Supplementary Table 4. Performance comparison with existing methods in dataset 1[24]

Algorithms	TP	FN	FP	TN	Accuracy	NA*	Repeat
Cancer-Finder	0	0	146	10839	98.67%	0	Repeat 1
	0	0	139	10846	98.73%	0	Repeat 2
	0	0	144	10841	98.69%	0	Repeat 3
	0	0	145	10840	98.68%	0	Repeat 4
	0	0	142	10843	98.71%	0	Repeat 5
Casee	0	0	8756	2229	20.29%	0	Repeat 1
	0	0	1756	9229	84.01%	0	Repeat 2
	0	0	5551	5434	49.47%	0	Repeat 3
	0	0	4677	6308	57.42%	0	Repeat 4
	0	0	5392	5593	50.91%	0	Repeat 5
CopyKAT	0	0	6590	3778	36.44%	617	Repeat 1
	0	0	6484	3884	37.46%	617	Repeat 2
	0	0	6614	3754	36.21%	617	Repeat 3
	0	0	6579	3789	36.55%	617	Repeat 4
	0	0	6603	3765	36.31%	617	Repeat 5
SCEVAN	0	0	3805	6625	63.52%	555	Repeat 1
	0	0	3805	6625	63.52%	555	Repeat 2
	0	0	3805	6625	63.52%	555	Repeat 3
	0	0	3805	6625	63.52%	555	Repeat 4
	0	0	3805	6625	63.52%	555	Repeat 5
Ikarus_default	0	0	0	10985	100.00%	0	Repeat 1
	0	0	0	10985	100.00%	0	Repeat 2
	0	0	0	10985	100.00%	0	Repeat 3
	0	0	0	10985	100.00%	0	Repeat 4
	0	0	0	10985	100.00%	0	Repeat 5
Ikarus_retrained	0	0	146	10839	98.67%	0	Repeat 1
	0	0	126	10859	98.85%	0	Repeat 2
	0	0	110	10875	99.00%	0	Repeat 3
	0	0	114	10871	98.96%	0	Repeat 4
	0	0	123	10862	98.88%	0	Repeat 5

*In the CopyKAT test, 'NA' represents the number of cells predicted to be 'not.defined' or discarded. In SCEVAN's tests, 'NA' indicates the number of cells that have been omitted from the calculation or marked as 'filtered' in the result file.

Supplementary Table 5. Performance comparison with existing methods in dataset 2[25]

Algorithms	TP	FN	FP	TN	Accuracy	NA*	Repeat
Cancer-Finder	4866	135	0	0	97.30%	0	Repeat 1
	4871	130	0	0	97.40%	0	Repeat 2
	4880	121	0	0	97.58%	0	Repeat 3
	4874	127	0	0	97.46%	0	Repeat 4
	4869	132	0	0	97.36%	0	Repeat 5
Casee	4129	872	0	0	82.56%	0	Repeat 1
	4046	955	0	0	80.90%	0	Repeat 2
	4319	682	0	0	86.36%	0	Repeat 3
	4227	774	0	0	84.52%	0	Repeat 4
	4358	643	0	0	87.14%	0	Repeat 5
CopyKAT	879	3257	0	0	21.25%	516	Repeat 1
	880	3256	0	0	21.28%	516	Repeat 2
	876	3260	0	0	21.18%	516	Repeat 3
	873	3263	0	0	21.11%	516	Repeat 4
	883	3253	0	0	21.35%	516	Repeat 5
SCEVAN	3417	769	0	0	81.63%	816	Repeat 1
	3417	769	0	0	81.63%	816	Repeat 2
	3417	769	0	0	81.63%	816	Repeat 3
	3417	769	0	0	81.63%	816	Repeat 4
	3417	769	0	0	81.63%	816	Repeat 5
Ikarus_retrained	637	4364	0	0	12.74%	0	Repeat 1
	634	4367	0	0	12.68%	0	Repeat 2
	645	4356	0	0	12.90%	0	Repeat 3
	670	4331	0	0	13.40%	0	Repeat 4
	595	4406	0	0	11.90%	0	Repeat 5

*In the CopyKAT test, 'NA' represents the number of cells predicted to be 'not.defined' or discarded. In SCEVAN's tests, 'NA' indicates the number of cells that have been omitted from the calculation or marked as 'filtered' in the result file.

Supplementary Table 6. Performance comparison with existing methods in dataset 3[26]

Algorithms	TP	FN	FP	TN	Similarity	NA*	Repeat
Cancer-Finder	34206	37	484	5219	98.70%	0	Repeat 1
	34208	35	484	5219	98.70%	0	Repeat 2
	34216	27	491	5212	98.70%	0	Repeat 3
	34212	31	488	5215	98.70%	0	Repeat 4
	34205	38	486	5217	98.69%	0	Repeat 5
Casee	28561	5682	3550	2153	76.89%	0	Repeat 1
	29675	4568	3926	1777	78.74%	0	Repeat 2
	28537	5706	3683	2020	76.50%	0	Repeat 3
	30029	4214	3880	1823	79.74%	0	Repeat 4
	26839	7404	3269	2434	73.28%	0	Repeat 5
CopyKAT	13323	18437	479	3561	47.16%	4146	Repeat 1
	11791	19969	1224	2816	40.80%	4146	Repeat 2
	12682	19078	1285	2755	43.12%	4146	Repeat 3
	12786	18974	1296	2744	43.38%	4146	Repeat 4
	13132	18628	469	3571	46.66%	4146	Repeat 5
SCEVAN	12358	20088	295	4105	44.68%	3100	Repeat 1
	12717	19729	296	4104	45.65%	3100	Repeat 2
	12722	19724	296	4104	45.67%	3100	Repeat 3
	12721	19725	285	4115	45.69%	3100	Repeat 4
	12357	20089	284	4116	44.71%	3100	Repeat 5
Ikarus_retrained	34237	6	577	5126	98.54%	0	Repeat 1
	34237	6	582	5121	98.53%	0	Repeat 2
	34238	5	578	5125	98.54%	0	Repeat 3
	34238	5	581	5122	98.53%	0	Repeat 4
	34240	3	590	5113	98.52%	0	Repeat 5

*In the CopyKAT test, 'NA' represents the number of cells predicted to be 'not.defined' or discarded. In SCEVAN's tests, 'NA' indicates the number of cells that have been omitted from the calculation or marked as 'filtered' in the result file.

Supplementary Table 7. Performance comparison with existing methods in dataset 4[27]

Algorithms	TP	FN	FP	TN	Similarity	NA*	Repeat
Cancer-Finder	52004	427	10102	4577	84.31%	0	Repeat 1
	52030	401	10194	4485	84.21%	0	Repeat 2
	52058	373	10237	4442	84.19%	0	Repeat 3
	52055	376	10334	4345	84.04%	0	Repeat 4
	52038	393	10232	4447	84.17%	0	Repeat 5
Casee	50969	1462	11659	3020	80.45%	0	Repeat 1
	50086	2345	10565	4114	80.76%	0	Repeat 2
	50715	1716	8723	5956	84.44%	0	Repeat 3
	51076	1355	11005	3674	81.58%	0	Repeat 4
	50777	1654	11934	2745	79.75%	0	Repeat 5
CopyKAT	29329	18318	7695	6472	57.92%	5296	Repeat 1
	30144	17503	8308	5859	58.24%	5296	Repeat 2
	30779	16868	8398	5769	59.13%	5296	Repeat 3
	35285	12362	8393	5774	66.42%	5296	Repeat 4
	30527	17120	7670	6497	59.90%	5296	Repeat 5
SCEVAN	37215	10966	7351	6967	70.69%	0	Repeat 1
	37215	10966	7351	6967	70.69%	0	Repeat 2
	37389	10792	7361	6957	70.95%	0	Repeat 3
	37123	11058	7316	7002	70.60%	0	Repeat 4
	37297	10884	7326	6992	70.86%	0	Repeat 5
Ikarus_default	47429	5002	14574	105	70.83%	0	Repeat 1
	47295	5136	14574	105	70.63%	0	Repeat 2
	47110	5321	14574	105	70.35%	0	Repeat 3
	47347	5084	14574	105	70.71%	0	Repeat 4
	47340	5091	14574	105	70.70%	0	Repeat 5
Ikarus_retrained	52424	7	11386	3293	83.02%	0	Repeat 1
	52427	4	12054	2625	82.03%	0	Repeat 2
	52425	6	11424	3255	82.97%	0	Repeat 3
	52427	4	12051	2628	82.04%	0	Repeat 4
	52423	8	11204	3475	83.29%	0	Repeat 5

*In the CopyKAT test, 'NA' represents the number of cells predicted to be 'not.defined' or discarded. In SCEVAN's tests, 'NA' indicates the number of cells that have been omitted from the calculation or marked as 'filtered' in the result file.

Supplementary Table 8. Performance comparison with existing methods in dataset 5[28,29]

Algorithms	TP	FN	FP	TN	Similarity	NA*	Repeat
Cancer-Finder	244	18	14	81	91.04%	0	Repeat 1
	244	18	14	81	91.04%	0	Repeat 2
	244	18	14	81	91.04%	0	Repeat 3
	244	18	15	80	90.76%	0	Repeat 4
	244	18	15	80	90.76%	0	Repeat 5
Casee	238	24	23	72	86.83%	0	Repeat 1
	244	18	40	55	83.75%	0	Repeat 2
	238	24	23	72	86.83%	0	Repeat 3
	241	21	23	72	87.68%	0	Repeat 4
	238	24	18	77	88.24%	0	Repeat 5
CopyKAT	108	152	0	94	57.06%	3	Repeat 1
	108	152	0	94	57.06%	3	Repeat 2
	108	152	0	94	57.06%	3	Repeat 3
	108	152	0	94	57.06%	3	Repeat 4
	108	152	0	94	57.06%	3	Repeat 5
SCEVAN	108	152	0	94	57.06%	3	Repeat 1
	108	152	0	94	57.06%	3	Repeat 2
	108	152	0	94	57.06%	3	Repeat 3
	108	152	0	94	57.06%	3	Repeat 4
	108	152	0	94	57.06%	3	Repeat 5
Ikarus_retrained	235	27	11	84	89.36%	0	Repeat 1
	235	27	11	84	89.36%	0	Repeat 2
	235	27	11	84	89.36%	0	Repeat 3
	235	27	11	84	89.36%	0	Repeat 4
	235	27	11	84	89.36%	0	Repeat 5

*In the CopyKAT test, 'NA' represents the number of cells predicted to be 'not.defined' or discarded. In SCEVAN's tests, 'NA' indicates the number of cells that have been omitted from the calculation or marked as 'filtered' in the result file.

Supplementary Table 9. Performance comparison with existing methods in dataset 6[30]

Algorithms	TP	FN	FP	TN	Similarity	NA*	Repeat
Cancer-Finder	10686	5549	648	27141	85.92%	0	Repeat 1
	10615	5620	645	27144	85.77%	0	Repeat 2
	10673	5562	667	27122	85.85%	0	Repeat 3
	10649	5586	661	27128	85.81%	0	Repeat 4
	10603	5632	638	27151	85.76%	0	Repeat 5
Casee	12164	4071	13161	14628	60.86%	0	Repeat 1
	11111	5124	11243	16546	62.82%	0	Repeat 2
	11281	4954	11695	16094	62.18%	0	Repeat 3
	13210	3025	15422	12367	58.10%	0	Repeat 4
	11331	4904	11985	15804	61.64%	0	Repeat 5
CopyKAT	3984	3199	6715	16626	67.52%	13500	Repeat 1
	3863	3320	6332	17009	68.38%	13500	Repeat 2
	3713	3470	5885	17456	69.35%	13500	Repeat 3
	3349	3834	6079	17262	67.52%	13500	Repeat 4
	3932	3251	6587	16754	67.77%	13500	Repeat 5
SCEVAN	3860	3670	6156	18174	69.16%	12164	Repeat 1
	3866	3664	6190	18140	69.07%	12164	Repeat 2
	3811	3719	7250	17080	65.57%	12164	Repeat 3
	3805	3725	7216	17114	65.66%	12164	Repeat 4
	3871	3659	6232	18098	68.95%	12164	Repeat 5
Ikarus_default	14507	1728	8209	19580	77.43%	0	Repeat 1
	14502	1733	8196	19593	77.45%	0	Repeat 2
	14503	1732	8196	19593	77.45%	0	Repeat 3
	14504	1731	8205	19584	77.43%	0	Repeat 4
	14505	1730	8201	19588	77.44%	0	Repeat 5
Ikarus_retrained	14280	1955	8402	19387	76.47%	0	Repeat 1
	14331	1904	7167	20622	79.40%	0	Repeat 2
	14356	1879	8476	19313	76.48%	0	Repeat 3
	14347	1888	7169	20620	79.43%	0	Repeat 4
	14335	1900	8383	19406	76.64%	0	Repeat 5

*In the CopyKAT test, 'NA' represents the number of cells predicted to be 'not.defined' or discarded. In SCEVAN's tests, 'NA' indicates the number of cells that have been omitted from the calculation or marked as 'filtered' in the result file and the number of cells in the **dataset 6** where Patient No. 40 terminated the run in SCEVAN, and did not output the result.

Supplementary Table 10. Performance comparison with existing methods in dataset 7[30]

Algorithms	TP	FN	FP	TN	Similarity	NA*	Repeat
Cancer-Finder	11200	2934	4231	26749	84.12%	0	Repeat 1
	11172	2962	4262	26718	83.99%	0	Repeat 2
	11190	2944	4306	26674	83.93%	0	Repeat 3
	11139	2995	4129	26851	84.21%	0	Repeat 4
	11156	2978	4205	26775	84.08%	0	Repeat 5
Casee	10028	4106	11891	19089	64.54%	0	Repeat 1
	9441	4693	10782	20198	65.70%	0	Repeat 2
	9470	4664	10280	20700	66.88%	0	Repeat 3
	9376	4758	10120	20860	67.02%	0	Repeat 4
	9880	4254	11428	19552	65.24%	0	Repeat 5
CopyKAT	5162	2704	4791	15132	73.03%	17325	Repeat 1
	5156	2710	4844	15079	72.82%	17325	Repeat 2
	6232	1634	2656	17267	84.56%	17325	Repeat 3
	6441	1425	2810	17113	84.76%	17325	Repeat 4
	6404	1462	3124	16799	83.50%	17325	Repeat 5
SCEVAN	6300	1784	3157	17813	82.99%	16060	Repeat 1
	6609	1475	3170	17800	84.01%	16060	Repeat 2
	6300	1784	3157	17813	82.99%	16060	Repeat 3
	6300	1784	3157	17813	82.99%	16060	Repeat 4
	6300	1784	3157	17813	82.99%	16060	Repeat 5
Ikarus_default	2169	11965	2	30978	73.47%	0	Repeat 1
	2173	11961	2	30978	73.48%	0	Repeat 2
	2173	11961	2	30978	73.48%	0	Repeat 3
	2175	11959	2	30978	73.49%	0	Repeat 4
	2174	11960	2	30978	73.48%	0	Repeat 5
Ikarus_retrained	6223	7911	44	30936	82.37%	0	Repeat 1
	6388	7746	55	30925	82.71%	0	Repeat 2
	6227	7907	43	30937	82.38%	0	Repeat 3
	6327	7807	49	30931	82.59%	0	Repeat 4
	6379	7755	52	30928	82.69%	0	Repeat 5

*In the CopyKAT test, 'NA' represents the number of cells predicted to be 'not.defined' or discarded. In SCEVAN's tests, 'NA' indicates the number of cells that have been omitted from the calculation or marked as 'filtered' in the result file.

Supplementary Table 11. Performance comparison with existing methods in dataset 8[30]

Algorithms	TP	FN	FP	TN	Similarity	NA*	Repeat
Cancer-Finder	9808	1295	4023	29558	88.10%	0	Repeat 1
	9774	1329	4059	29522	87.94%	0	Repeat 2
	9796	1307	4083	29498	87.94%	0	Repeat 3
	9763	1340	3966	29615	88.13%	0	Repeat 4
	9769	1334	4026	29555	88.00%	0	Repeat 5
Casee	7938	3165	11468	22113	67.25%	0	Repeat 1
	8071	3032	12658	20923	64.89%	0	Repeat 2
	7471	3632	9827	23754	69.88%	0	Repeat 3
	8682	2421	13660	19921	64.01%	0	Repeat 4
	8900	2203	15566	18015	60.23%	0	Repeat 5
CopyKAT	3563	1312	4385	20077	80.58%	15347	Repeat 1
	3334	1541	5086	19376	77.41%	15347	Repeat 2
	3219	1656	7191	17271	69.84%	15347	Repeat 3
	3599	1276	4695	19767	79.65%	15347	Repeat 4
	2986	1889	8589	15873	64.28%	15347	Repeat 5
SCEVAN	3663	1392	4985	20976	79.44%	13668	Repeat 1
	3663	1392	4985	20976	79.44%	13668	Repeat 2
	3663	1392	4985	20976	79.44%	13668	Repeat 3
	3663	1392	4985	20976	79.44%	13668	Repeat 4
	3663	1392	4985	20976	79.44%	13668	Repeat 5
Ikarus_default	9917	1186	12319	21262	69.78%	0	Repeat 1
	9917	1186	12318	21263	69.78%	0	Repeat 2
	9941	1162	12322	21259	69.82%	0	Repeat 3
	9917	1186	12317	21264	69.78%	0	Repeat 4
	9883	1220	12321	21260	69.70%	0	Repeat 5
Ikarus_retrained	10652	451	13002	20579	69.89%	0	Repeat 1
	10657	446	13017	20564	69.87%	0	Repeat 2
	10599	504	12881	20700	70.05%	0	Repeat 3
	10603	500	12891	20690	70.03%	0	Repeat 4
	10605	498	12792	20789	70.26%	0	Repeat 5

*In the CopyKAT test, 'NA' represents the number of cells predicted to be 'not.defined' or discarded. In SCEVAN's tests, 'NA' indicates the number of cells that have been omitted from the calculation or marked as 'filtered' in the result file.

Supplementary Table 12. Performance comparison with existing methods in dataset 9[30]

Algorithms	TP	FN	FP	TN	Similarity	NA*	Repeat
Cancer-Finder	11844	468	5143	76120	94.00%	0	Repeat 1
	11844	468	5082	76181	94.07%	0	Repeat 2
	11840	472	5085	76178	94.06%	0	Repeat 3
	11820	492	4978	76285	94.15%	0	Repeat 4
	11838	474	5050	76213	94.10%	0	Repeat 5
Casee	11054	1258	41530	39733	54.27%	0	Repeat 1
	10600	1712	32183	49080	63.78%	0	Repeat 2
	10672	1640	34758	46505	61.10%	0	Repeat 3
	10849	1463	34928	46335	61.11%	0	Repeat 4
	10444	1868	32572	48691	63.20%	0	Repeat 5
CopyKAT	5627	1278	15052	34652	71.15%	36966	Repeat 1
	5632	1273	7339	42365	84.79%	36966	Repeat 2
	5539	1366	6164	43540	86.70%	36966	Repeat 3
	5697	1208	8495	41209	82.86%	36966	Repeat 4
	5454	1451	12525	37179	75.31%	36966	Repeat 5
SCEVAN	6268	855	15147	37277	73.13%	34028	Repeat 1
	6271	852	15145	37279	73.14%	34028	Repeat 2
	6271	852	15145	37279	73.14%	34028	Repeat 3
	6268	855	15147	37277	73.13%	34028	Repeat 4
	6268	855	15147	37277	73.13%	34028	Repeat 5
Ikarus_default	10221	2091	176	81087	97.58%	0	Repeat 1
	10218	2094	175	81088	97.58%	0	Repeat 2
	10209	2103	176	81087	97.56%	0	Repeat 3
	10207	2105	174	81089	97.56%	0	Repeat 4
	10221	2091	175	81088	97.58%	0	Repeat 5
Ikarus_retrained	11890	422	7544	73719	91.49%	0	Repeat 1
	11916	396	7747	73516	91.30%	0	Repeat 2
	11896	416	7563	73700	91.47%	0	Repeat 3
	11911	401	7676	73587	91.37%	0	Repeat 4
	11860	452	7350	73913	91.66%	0	Repeat 5

*In the CopyKAT test, 'NA' represents the number of cells predicted to be 'not.defined' or discarded. In SCEVAN's tests, 'NA' indicates the number of cells that have been omitted from the calculation or marked as 'filtered' in the result file.

Supplementary Table 13. Performance comparison with existing methods in dataset 10[31]

Algorithms	TP	FN	FP	TN	Similarity	NA*	Repeat
Cancer-Finder	0	0	150	56322	99.73%	0	Repeat 1
	0	0	141	56331	99.75%	0	Repeat 2
	0	0	150	56322	99.73%	0	Repeat 3
	0	0	153	56319	99.73%	0	Repeat 4
	0	0	143	56329	99.75%	0	Repeat 5
Casee	0	0	31103	25369	44.92%	0	Repeat 1
	0	0	18970	37502	66.41%	0	Repeat 2
	0	0	19543	36929	65.39%	0	Repeat 3
	0	0	11892	44580	78.94%	0	Repeat 4
	0	0	14139	42333	74.96%	0	Repeat 5
CopyKAT	0	0	18916	32935	63.52%	4621	Repeat 1
	0	0	25718	26133	50.40%	4621	Repeat 2
	0	0	24015	27836	53.68%	4621	Repeat 3
	0	0	25669	26182	50.49%	4621	Repeat 4
	0	0	31201	20650	39.83%	4621	Repeat 5
SCEVAN	0	0	18284	26704	59.36%	11484	Repeat 1
	0	0	19108	25880	57.53%	11484	Repeat 2
	0	0	18556	26432	58.75%	11484	Repeat 3
	0	0	18897	26091	58.00%	11484	Repeat 4
	0	0	18563	26425	58.74%	11484	Repeat 5
Ikarus_default	0	0	0	56472	100.00%	0	Repeat 1
	0	0	0	56472	100.00%	0	Repeat 2
	0	0	0	56472	100.00%	0	Repeat 3
	0	0	0	56472	100.00%	0	Repeat 4
	0	0	0	56472	100.00%	0	Repeat 5
Ikarus_retrained	0	0	2	56470	100.00%	0	Repeat 1
	0	0	2	56470	100.00%	0	Repeat 2
	0	0	1	56471	100.00%	0	Repeat 3
	0	0	2	56470	100.00%	0	Repeat 4
	0	0	1	56471	100.00%	0	Repeat 5

*In the CopyKAT test, 'NA' represents the number of cells predicted to be 'not.defined' or discarded. In SCEVAN's tests, 'NA' indicates the number of cells that have been omitted from the calculation or marked as 'filtered' in the result file.

Supplementary Table 14. Memory consumption comparison of five algorithms

Number of cells / Algorithms	100	1,000	10,000	100,000	1,000,000
Cancer-Finder	13,532 KB	13,568 KB	3,924,116 KB	13,956,016 KB	14,865,940 KB
SCEVAN	1,657,160 KB	3,751,188 KB	NA	NA	NA
CaSee	12,878,114 KB	13,429,636 KB	21,388,220 KB	118,324,804 KB	120,300,268 KB
CopyKAT	707,728 KB	3,871,888 KB	25,668,260 KB	NA	NA
ikarus	298,576 KB	1,113,896 KB	12,877,444 KB	165,325,312 KB	165,368,696 KB

'NA' indicates that the method could not run correctly on the data.

Supplementary Table 15. Full name of cancer types

Cancer type	Full name of cancer types	Up-regulated genes in malignant cells
GBM	Glioblastoma multiforme	*BEX3,MARCKSL1,SOX2,NOVA1,TUBB2B,CKB,PTN,GPM6B,MAP2,UCHL1*
PDAC	Pancreatic ductal adenocarcinoma	*TPM1,MDK,KRT18,EPCAM,SMIM22,DSTN,S100A13,CYSTM1,CD59,KRT8*
LUAD	Lung adenocarcinoma	*GSTP1,SPINT2,MGST1,CD9,KRT18,KRT8,S100A13,RAB13,LGALS3BP, DSTN*
NSCLC	Non-small cell lung carcinoma	*SLC34A2,ELF3,SFTA2,NAPSA,CXCL17,AGR2,ATP1B1,CEACAM6,SOX4, MUC1*
CRC	Colorectal cancer	*IFI27,KRT8,KRT18,S100A16,MDK,LGALS4,PDLIM1,C19orf33,PHGR1, KRT19*
MCC	Merkel cell carcinoma	*TFAP2A,SOX2,BEX1,NHLH1,ISL1,EPCAM,TUBB2B,UCHL1,POU4F3,PKIB*
OV	Ovarian cancer	*TNS4,LHX1,ZNF608,SHROOM3,LCP1,ZBED3,ELF3,E2F5,PLPP3,SCEL*
ATC	Anaplastic thyroid carcinoma	*CALD1,COL6A2,TCEAL9,GNG11,MAP1B,CTHRC1,RAI14,MARCKS,SGCE,FAM114A1*
HCC	Hepatocellular carcinoma	*ITM2C,MZB1,STMN1,HMGN1,HSP90AB1,PEBP1,TSPAN13,HMGA1,PLD4,HLA-DQA1*
AA	Aplastic anemia	*PTPRZ1,MIR9-1HG,FABP7,BCAN,MT3,CKB,GPM6B,SLC1A2,TSC22D4,RHOBTB3*
TNBC	Triple-negative breast cancer	*KRT8,KRT7,CNN3,KRT19,MGST1,CD24,EPCAM,SOX4,ELF3,PFN2*
UCEC	Uterine corpus endometrial carcinoma	*DSP,CNN3,CDH1,KLF5,PRSS8,PKP3,LSR,KRT18,VTCN1,DMKN*
LUSC	Lung squamous cell carcinoma	*CD9,HSPB1,GSTP1,KRT19,BEX3,CD59,KRT18,PDLIM1,LGALS3BP,KRT8*
NBL	Neuroblastoma	*CHGB,TPH1,TTR,PHGR1,SYT13,CHGA,TAC1,PCSK1,SERPINA1,PCSK1N*
MIUBC	Muscle-invasive urothelial bladder cancer	*PRKCDBP,CALD1,PPIC,PTRF,APP,PLS3,NGFRAP1,TPM1,MXRA8,PHLDA3*
STAD	Stomach adenocarcinoma	*SERINC2,KLF5,CLDN3,CXADR,LAD1,PPP1R1B,SMIM22,FHL2,EPCAM, GMDS*
DCIS	Ductal carcinoma in situ	*ANKRD30A,FXYD3,EPCAM,IRX3,SIX1,SPDEF,RERG,MYO6,CHMP4C, GATA3*
BCC	Basal cell carcinoma	*CD9,CALD1,CAV1,SPARC,DST,APOE,SOX4,APP,GSTP1,KRT5*

Gene names are formatted in italics.

Supplementary Table 16. Information on the spatial transcriptome data

Slide	Publishes	Cancer Type	Number of Malignant spot	Malignant rate	Platform
HCC-1L	Rui, W. et al.[32]	hepatocellular carcinoma	969	38.59%	10x Visium
HCC-2L	Rui, W. et al.[32]	hepatocellular carcinoma	2036	49.78%	10x Visium
HCC-3L	Rui, W. et al.[32]	hepatocellular carcinoma	1229	26.97%	10x Visium
HCC-4L	Rui, W. et al.[32]	hepatocellular carcinoma	2036	51.83%	10x Visium
CRC-1	Qi, J. et al[33]	colorectal cancer	963	25.72%	10x Visium
CRC-2	Qi, J. et al[33]	colorectal cancer	844	26.69%	10x Visium
CRC-3	Qi, J. et al[33]	colorectal cancer	502	34.10%	10x Visium
ICC	Rui, W. et al[32]	intrahepatic cholangiocarcinoma	1468	35.51%	10x Visium
OV	10x Company Website	ovarian cancer	1822	67.68%	10x Visium
BRCA-1	10x Company Website	breast cancer	2378	71.82%	10x Visium
BRCA-2	10x Company Website	breast cancer	2208	55.13%	10x Visium
RCC-1	Meylan, M. et al.[34]	renal call carcinoma	872	43.97%	10x Visium
RCC-2	Meylan, M. et al.[34]	renal call carcinoma	928	68.79%	10x Visium
RCC-3	Meylan, M. et al.[34]	renal call carcinoma	1215	60.54%	10x Visium

Supplementary Table 17. Evaluation results of Domain Generalization strategies from Wang et al.[21]

PACS dataset (ResNet-18)		PACS dataset (ResNet-50)		Home-Office dataset (ResNet-18)		Home-Office dataset (ResNet-50)	
Methods	Result	Methods	Result	Methods	Result	Methods	Result
V-REx[7]	83.85	Mixup	88.18	Mixup	64.33	Mixup	71.24
RSC[35]	83.6	CORAL	87.9	MMD	64.15	V-REx	70.81
DANN[36,37]	83.57	V-REx	87.75	CORAL	63.97	ERM	70.44
MMD[38]	83.21	DANN	87.6	GroupDRO	63.92	GroupDRO	70.39
CORAL[39]	82.83	MMD	87.07	V-REx	63.84	MMD	70.36
ERM	82.75	RSC	86.85	ERM	63.54	CORAL	70.27
Mixup[40]	82.26	GroupDRO	86.84	RSC	63.35	DANN	70.02
GroupDRO[41]	82.19	ERM	86.31	DANN	62.57	RSC	69.36
ANDMask[42]	80.47	ANDMask	85.07	ANDMask	60.69	ANDMask	67.59

These results were gathered from https://github.com/jindongwang/transferlearning/tree/master/code/DeepDG. The results of one strategy (DIFEX[43]) were discarded because it was evaluated only in one experiment and not in the other three experiments.

Supplementary Figures

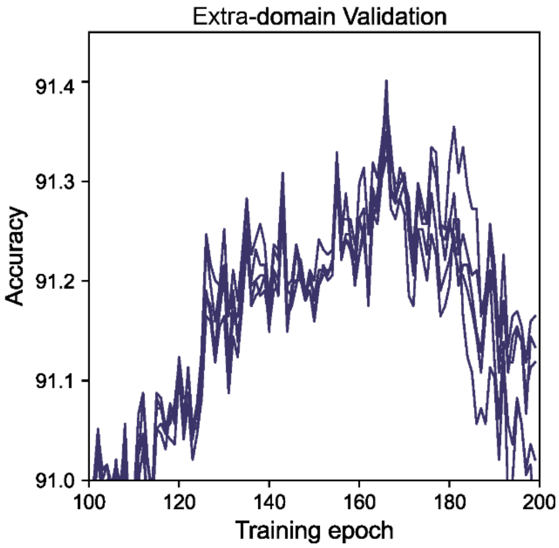

Supplementary Figure 1 | Accuracy changes in breast cancer data across training epochs. When the accuracy of the breast cancer data (36,201 cells with 17,864 malignant cells) is maximized, the model's training was terminated and it was employed for subsequent predictive analysis. Each line represents a training session. In five separate replicates, the model was optimized in 176, 166, 166 166, and 166 epochs (*n*=36,201 cells examined over 5 independent experiments). Source data are provided as a Source Data file.

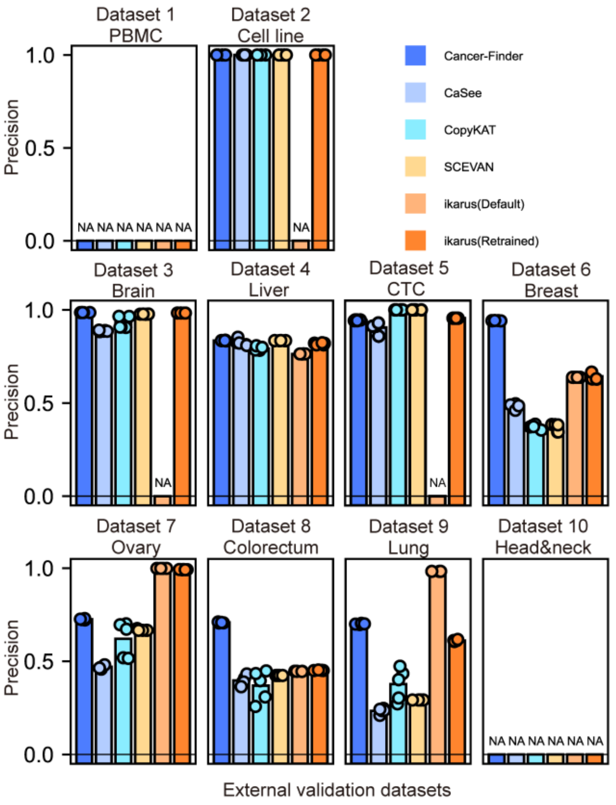

Supplementary Figure 2 | Comparison of the Precision of Cancer-Finder and four other cell annotation algorithms based on external validation datasets. Since most of these available algorithms exhibit some level of randomness in their results across runs, all tests were conducted in parallel five times. It is noteworthy that the pre-trained Cancer-Finder consistently yields uniform predictions on the external datasets. Recognizing that variations in the training process and data may introduce a degree of randomness, we conducted five training sessions for Cancer-Finder here, completing the specified 5 independent and repeated experiments (detailed in **Supplementary Note 2**). The detailed cell numbers (*n* numbers) and malignancy percentages for each dataset are shown in **Figure 3a**. For ikarus (retrained), we employed the same strategy. The presence of an 'NA' denotes that the method returns an error and cannot be executed with these data, or that the dataset contains only positive or negative samples, therefore the indicator cannot be calculated. Source data are provided as a Source Data file.

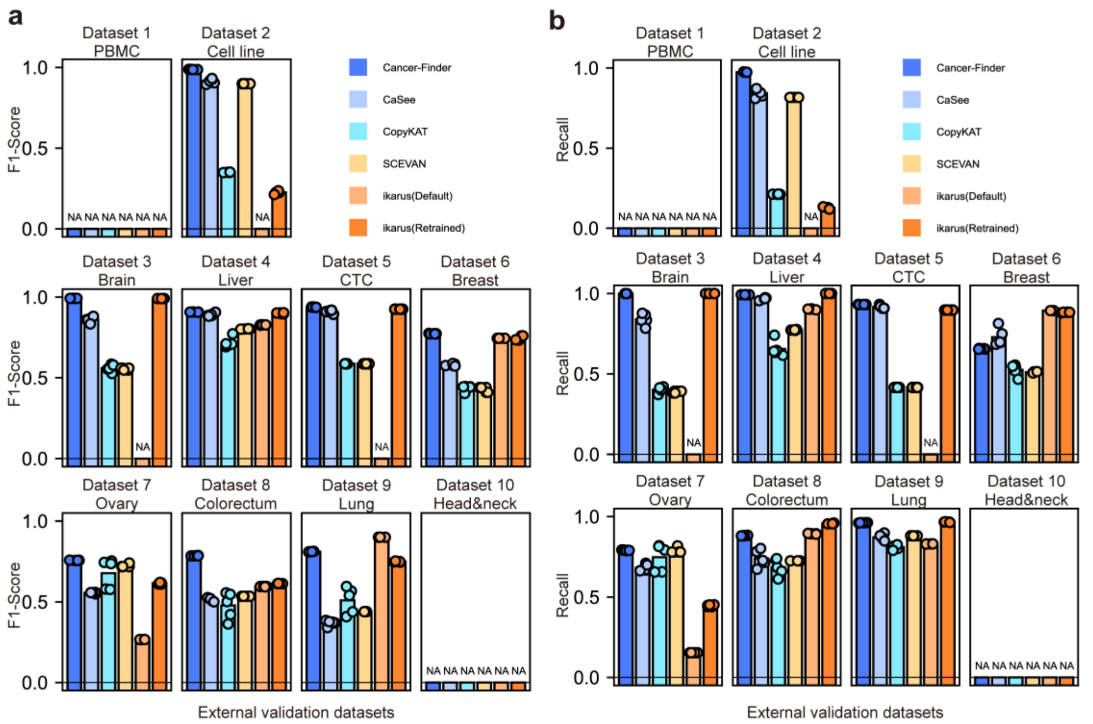

Supplementary Figure 3 | Comparison of the F1-Scores and the recall rates of Cancer-Finder and four other cell annotation algorithms based on external validation datasets. a, Comparison of Cancer-Finder's F1-Scores to four other cell annotation algorithms on 10 external validation datasets. b, Comparison of Cancer-Finder's recall rates to four other cell annotation algorithms on 10 external validation datasets. Since most of these available algorithms exhibit some level of randomness in their results across runs, all tests were conducted in parallel five times. It is noteworthy that the pre-trained Cancer-Finder consistently yields uniform predictions on the external datasets. Recognizing that variations in the training process and data may introduce a degree of randomness, we conducted five training sessions for Cancser-Finder here, completing the specified 5 independent and repeated experiments (detailed in **Supplementary Note 2**). The detailed cell numbers (*n* numbers) and malignancy percentages for each dataset are shown in **Figure 3a**. For ikarus (retrained), we employed the same strategy. The presence of an ‘NA’ denotes that the method returns an error and cannot be executed with these data, or that the dataset contains only positive or negative samples, therefore the indicator cannot be calculated. Source data are provided as a Source Data file.

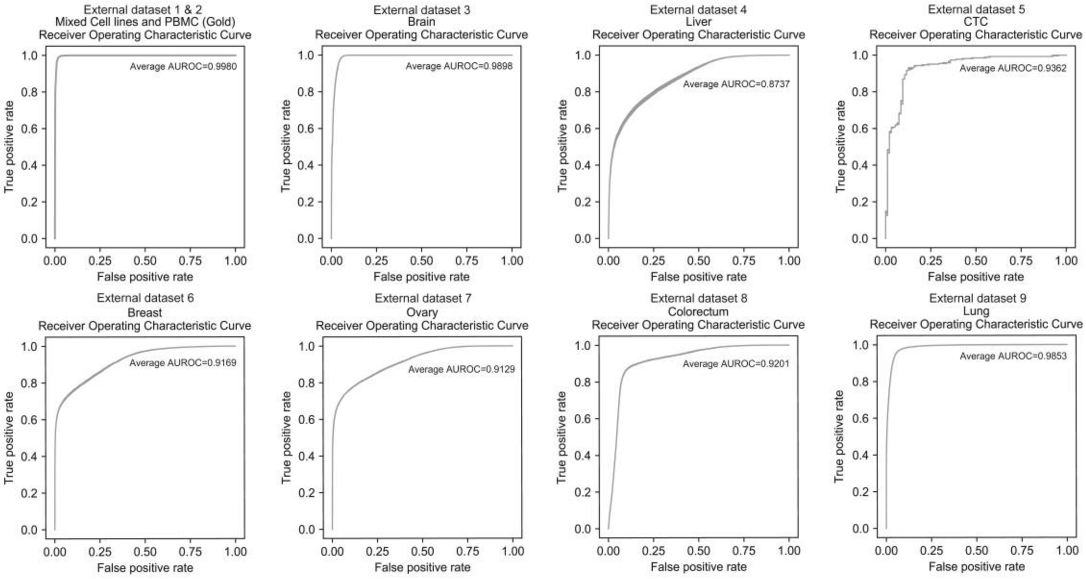

Supplementary Figure 4 | AUROC of Cancer-Finder's prediction results based on external validation datasets. Since most of these available algorithms exhibit some level of randomness in their results across runs, all tests were conducted in parallel five times. It is noteworthy that the pre-trained Cancer- Finder consistently yields uniform predictions on the external datasets. Recognizing that variations in the training process and data may introduce a degree of randomness, we conducted five training sessions for Cancer-Finder here, completing the specified 5 independent and repeated experiments (detailed in **Supplementary Note 2**). Each line represents the result of one training session. The detailed cell numbers (*n* numbers) and malignancy percentages for each dataset are shown in **Figure 3a**. For ikarus (retrained), we employed the same strategy. The average AUROC was presented. Source data are provided as a Source Data file.

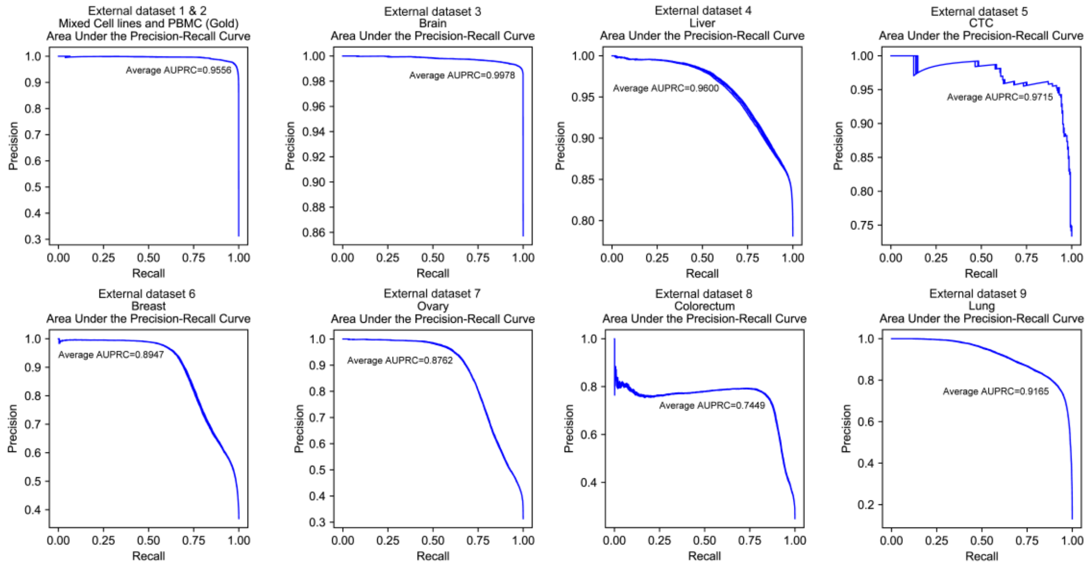

Supplementary Figure 5 | AUPRC of Cancer-Finder's prediction results based on external validation datasets. Since most of these available algorithms exhibit some level of randomness in their results across runs, all tests were conducted in parallel five times. It is noteworthy that the pre-trained Cancer- Finder consistently yields uniform predictions on the external datasets. Recognizing that variations in the training process and data may introduce a degree of randomness, we conducted five training sessions for Cancer-Finder here, completing the specified 5 independent and repeated experiments (detailed in **Supplementary Note 2**). Each line represents the result of one training session. The detailed cell numbers (*n* numbers) and malignancy percentages for each dataset are shown in **Figure 3a**. For ikarus (retrained), we employed the same strategy. The average AUPRC was presented. Source data are provided as **Supplementary Data 1**.

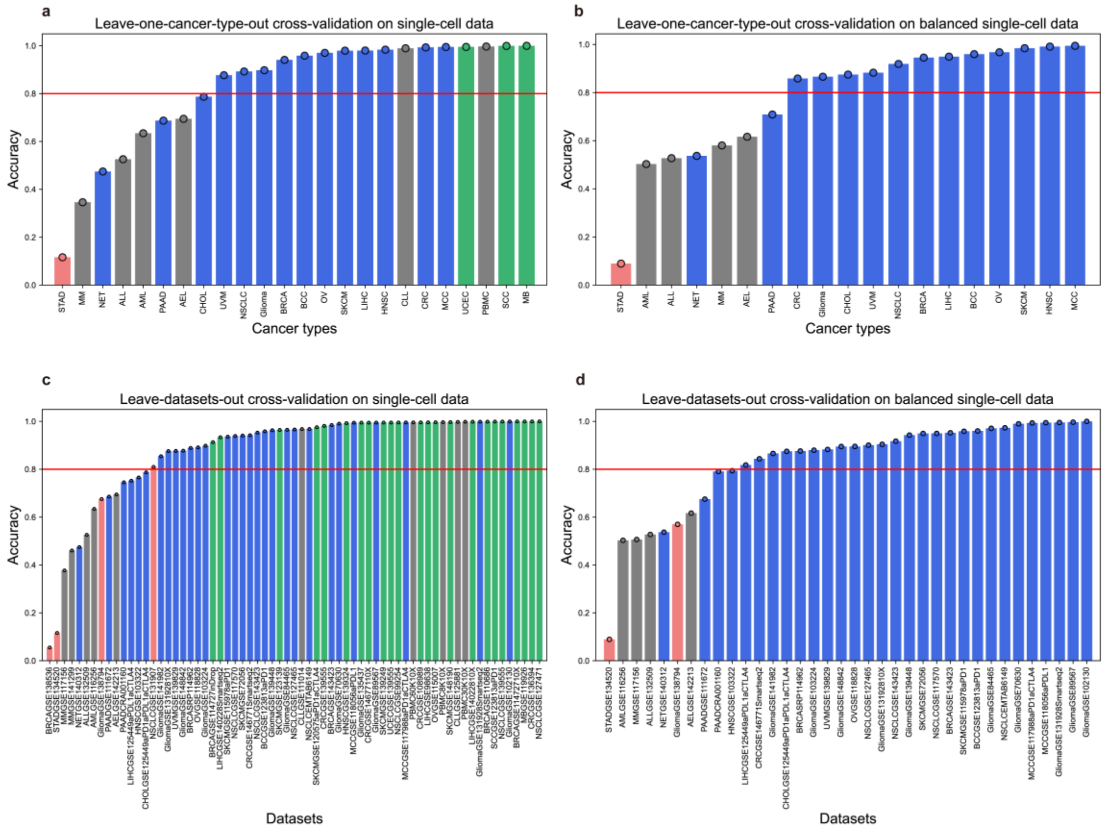

Supplementary Figure 6 | Results of leave-one-cancer-type-out and leave-datasets-out cross-validations. a,b Results of leave-one-cancer-type-out cross-validation on single-cell data and balanced single-cell data. Leave-one-cancer-type-out cross-validation was performed by excluding scRNA-seq data from one cancer, training Cancer-Finder with data from other cancers, and predicting cell annotation labels for the excluded cancer. Cancer-Finder performs well (accuracy > 0.8) on most cancers, but its performance is limited on hematologic tumors (colored in grey), possibly due to the significant difference between hematologic and solid tumors. Notably, a discrepancy was discovered between the original study[8] and TISCH's annotation on cancer with the lowest accuracy (colored red), which may have been caused by database collection errors in the database. c,d Results of leave-datasets-out cross-validation on single-cell data and balanced single-cell data. Leave-datasets-out cross-validation was conducted by excluding one scRNA-seq dataset at a time, training Cancer-Finder with data from other datasets, and predicting cell annotation labels for the excluded dataset. On four datasets (colored red), discrepancies were discovered between the original study[44,8,45,46] and TISCH's annotation, possibly due to database collection errors. These results demonstrated that Cancer-Finder can be a useful aid for relevant database annotation and error detection. The datasets represented by the green bars consist of either all-malignant or all-non-malignant cells and are therefore not included in the balanced single-cell data. Other datasets are colored in blue. Source data are provided as a Source Data file.

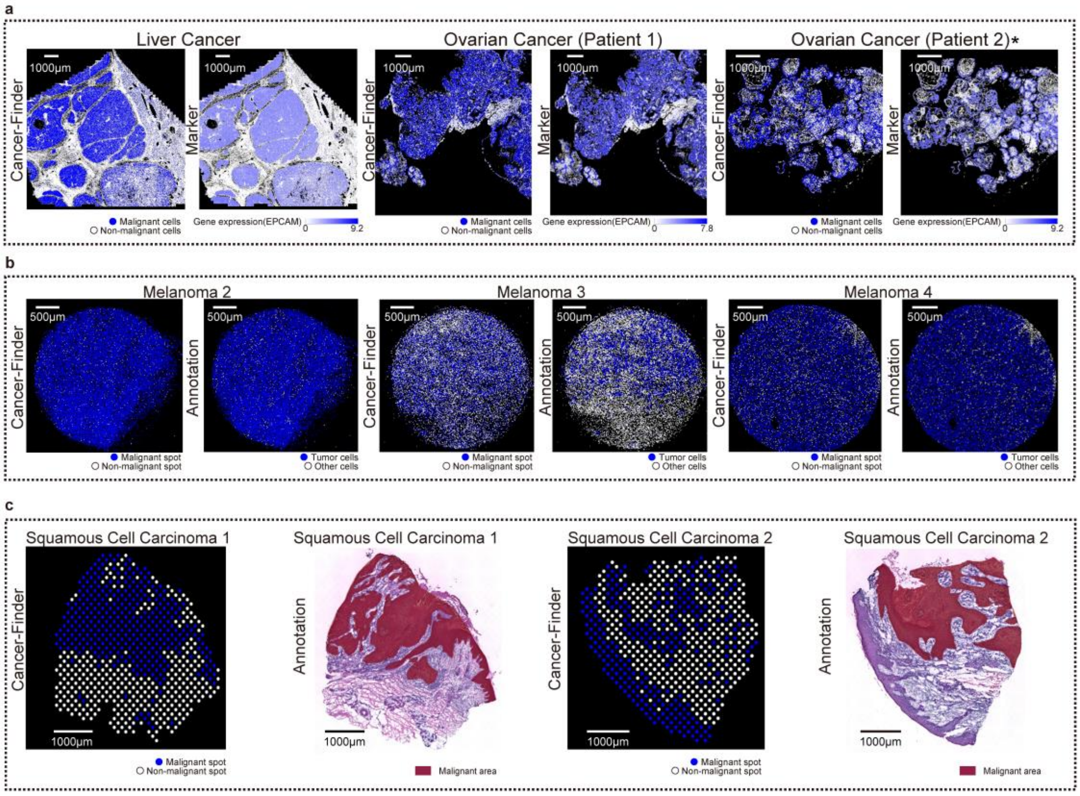

Supplementary Figure 7 | Application expansion of Cancer-Finder. a, Performance of Cancer-Finder on MERFISH data. Here, the optimal Softmax threshold (threshold = 0.9766) was determined according to the ROC curve based on an external MERFISH slide. '*' denotes the slide used to determine the threshold value. b, Performance of Cancer-Finder on slide-seq data. c, Performance of Cancer-Finder on legacy ST data. Source data are provided as a Source Data file.

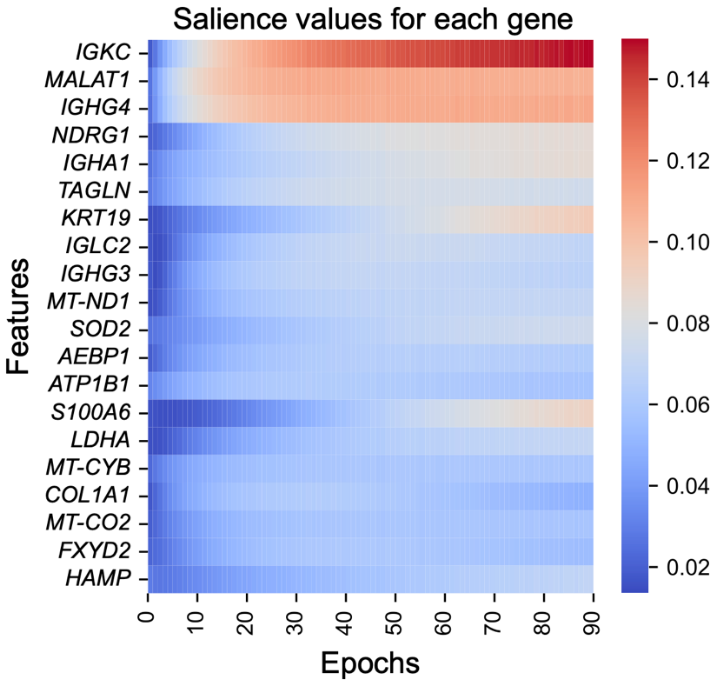

Supplementary Figure 8 | Changes in the salience value of features during training. Here, the salience values for the top 20 genes are displayed. Gene names are formatted in italics. Source data are provided as a Source Data file.

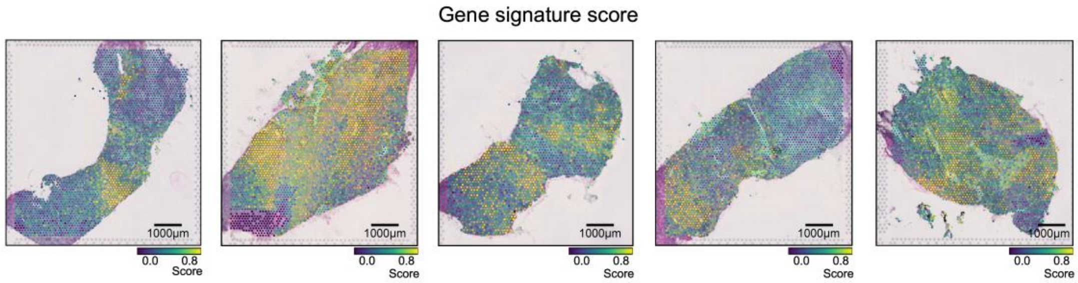

Supplementary Figure 9 | Gene signature score in 5 ccRCC slides. Here, the ssGESA scores in 5 ccRCC slides of the gene signature from Cancer-Finder are shown. Source data are provided as a Source Data file.

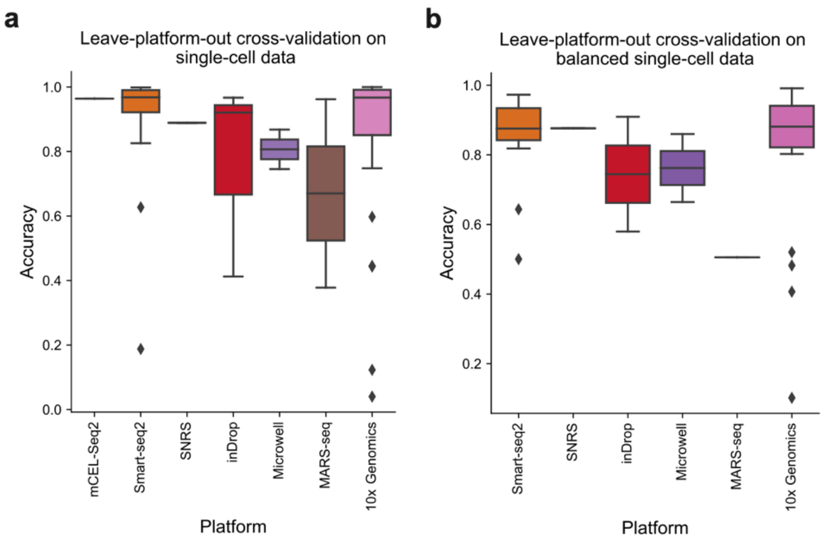

Supplementary Figure 10 | Results of leave-platform-out cross-validations. Leave-platform-out cross-validation was performed by excluding datasets from one platform, training Cancer-Finder with data from other platforms, and predicting cell annotation labels for the excluded datasets. Each point represents the accuracy of a dataset. The dataset from mCEL-seq2 was excluded from the balanced validation because it contains only non-malignant (negative) cells. Notably, several datasets were excluded from balanced validation because they contained only all-malignant or all-nonmalignant cells. For the data presented in (a), the displayed platforms are mCEL-Seq2 (*n*=1), Smart-seq2 (*n*=18), SNRS (*n*=1), inDrop (*n*=3), Microwell (*n*=2), MARS-seq (*n*=2), and 10x_Genomics (*n*=41). For the data presented in (b), the displayed platforms are Smart-seq2 (*n*=11), SNRS (*n*=1), inDrop (*n*=1), Microwell (*n*=2), MARS-seq (*n*=1), and 10x_Genomics (*n*=19). The boxes are centered at median values, where the range of boxes represents the interquartile range (IQR) bounded by the first quartile (Q1) and the third quartile (Q3). Source data are provided as a Source Data file.

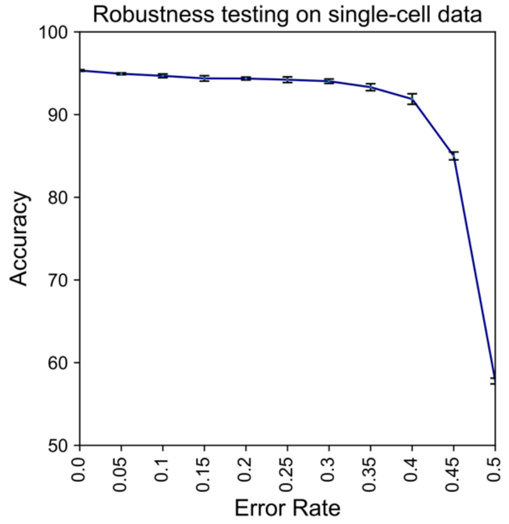

Supplementary Figure 11 | Performance of Cancer-Finder when the training set is partially incorrect. Here, we demonstrated that the overall performance of Cancer-

Finder remains stable in the presence of less than 35% incorrect labels by modifying the labels in the training set to incorrect annotations and then using them to train the model. Five-fold leave-cells-out cross-validation was performed for each rate (Error bars show mean ± standard deviation of these 5 validations, n=101,847 cells examined over 5 independent experiments). Source data are provided as a Source Data file.

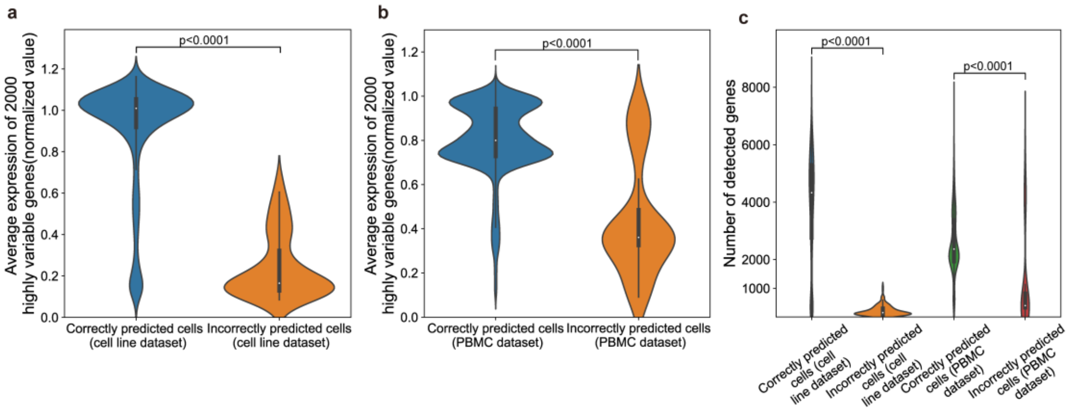

Supplementary Figure 12 | Comparisons between cells that were correctly and incorrectly predicted. Two gold standard datasets were used. a, Comparison of average expression of HVGs in correctly predicted and incorrectly predicted cells in the cell line dataset. Here, we use the top 2000 HVGs according to expression variance (n=5001, ****p<0.0001, paired t-tests). b, Comparison of average expression of HVGs in correctly predicted and incorrectly predicted cells in PBMC dataset. Here, we use the top 2000 HVGs according to expression variance (n=10985, ****p<0.0001, paired t-tests). c, Comparison of number of detected genes in correctly predicted and incorrectly predicted cells. Paired t-tests were used for the cell line dataset (n=5001, ****p<0.0001) and the PBMC dataset (n=5001, ****p<0.0001). The violins are centered at median values, where the range of violins represents the interquartile range (IQR) bounded by the first quartile (Q1) and the third quartile (Q3). Source data are provided as a Source Data file.

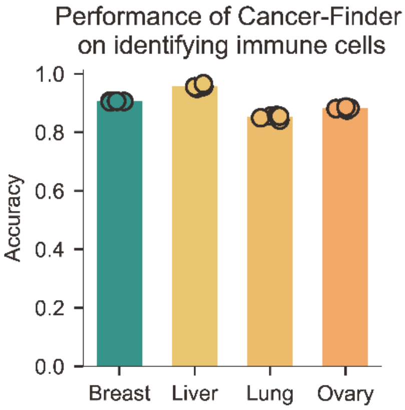

Supplementary Figure 13 | Performance of Cancer-Finder on identifying immune cells. Breast, lung, and ovary data are from Qian, J. et al[30], and liver data is from Bondoc,

A. et al[27]. The average accuracy on immune cell prediction reached 90.59% (Breast, *n*=44,024), 95.76% (Liver, *n*=67,110), 85.21% (Lung, *n*=93,575) and 88.18% (Ovary, *n*=45,114), respectively (Error bars show mean ± standard deviation of these 5 validations). Independent experiments were repeated 5 times. Source data are provided as a Source Data file.

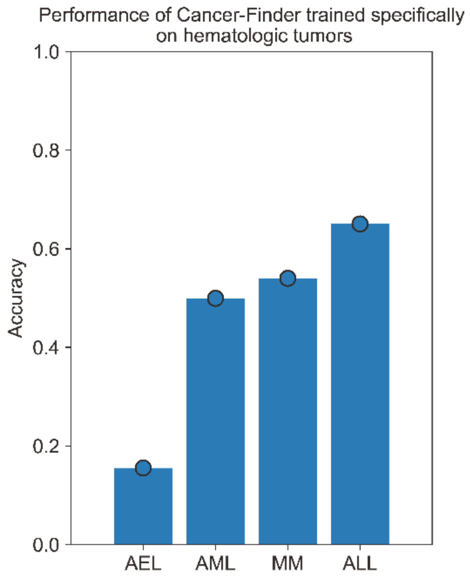

Supplementary Figure 14 | Performance of Cancer-Finder trained specifically on hematologic tumors. Here, we evaluate the model's accuracy on four hematologic cancers using the leave-one-cancer-type-out strategy. Source data are provided as a Source Data file.

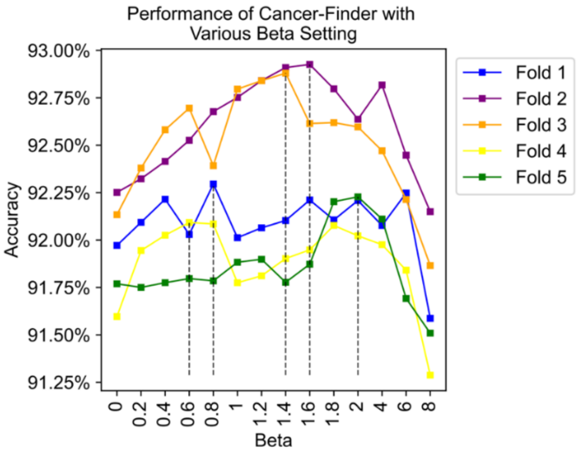

Supplementary Figure 15 | 5-fold cross-validation of Cancer-Finder with various β. Source data are provided as a Source Data file.

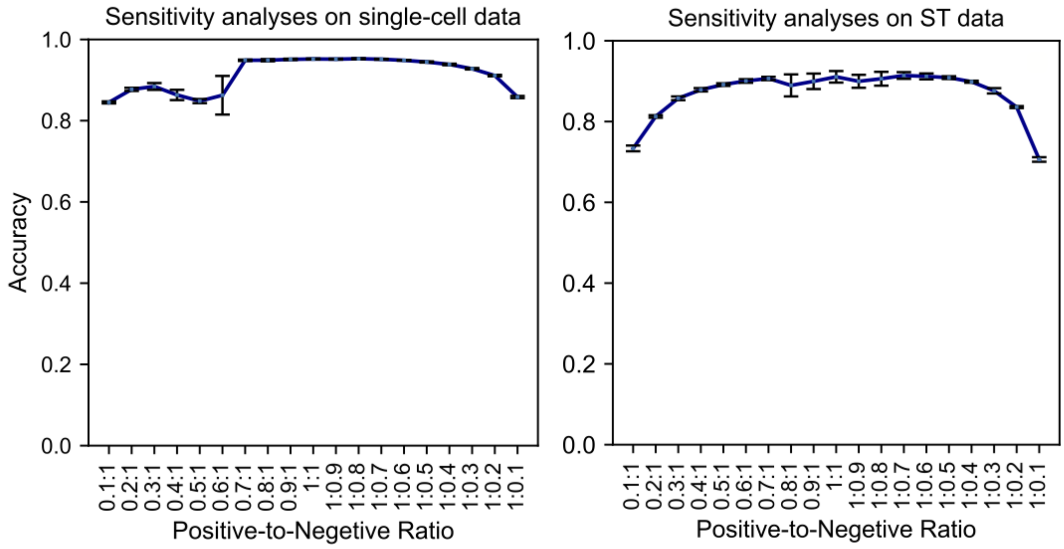

Supplementary Figure 16 | Sensitivity analysis on the ratio of positive to negative samples. Malignant or non-malignant cells (or spots) were sampled by down-sampling in each domain to produce a series of data with ratios ranging from 0.1:1 to 1:0.1. Then, 5-fold leave-cells-out cross-validations were performed to assess the performance of Cancer-Finder (Error bars show mean ± standard deviation of these 5 validations, *n*=101,847 cells examined over 5 independent experiments). Source data are provided as a Source Data file.

References

1. Simonyan, K., Vedaldi, A. & Zisserman, A. Deep Inside Convolutional Networks: Visualising Image Classification Models and Saliency Maps. in *International Conference on Learning Representations* (ICLR, 2014).
2. Sun, D. et al. TISCH: a comprehensive web resource enabling interactive single-cell transcriptome visualization of tumor microenvironment. *Nucleic Acids Res.* **49**, D1420–D1430 (2021).
3. Qi, J. et al. Single-cell and spatial analysis reveal interaction of FAP+ fibroblasts and SPP1+ macrophages in colorectal cancer. *Nat. Commun.* **13**, 1742 (2022).
4. Wu R. et al. Comprehensive analysis of spatial architecture in primary liver cancer. *Sci. Adv.* **7**, eabg3750 (2021).
5. Meylan, M. et al. Tertiary lymphoid structures generate and propagate anti-tumor antibody-producing plasma cells in renal cell cancer. *Immunity* **55**, 527-541.e5 (2022).
6. Xun, Z. et al. Reconstruction of the tumor spatial microenvironment along the malignant-boundary-nonmalignant axis. *Nat. Commun.* **14**, 933 (2023).
7. Krueger, D. et al. Out-of-Distribution Generalization via Risk Extrapolation. in *38th International Conference on Machine Learning* (ICML, 2021).
8. Zhang, P. et al. Dissecting the Single-Cell Transcriptome Network Underlying Gastric Premalignant Lesions and Early Gastric Cancer. *Cell Rep.* **30**, 4317 (2020).
9. Dohmen, J. et al. Identifying tumor cells at the single-cell level using machine learning. *Genome Biol.* **23**, 123 (2022).
10. Gao, R. et al. Delineating copy number and clonal substructure in human tumors from single-cell transcriptomes. *Nat. Biotechnol.* **39**, 599–608 (2021).
11. Sh, Y. et al. CaSee: A lightning transfer-learning model directly used to discriminate cancer/normal cells from scRNA-seq. *Oncogene* **41**, 4866–4876 (2022).
12. De Falco, A., Caruso, F., Su, X.-D., Iavarone, A. & Ceccarelli, M. A variational algorithm to detect the clonal copy number substructure of tumors from scRNA-seq data. *Nat. Commun.* **14**, 1074 (2023).
13. Chen, K. H., Boettiger, A. N., Moffitt, J. R., Wang, S. & Zhuang, X. Spatially resolved, highly multiplexed RNA profiling in single cells. *Science* **348**, aaa6090 (2015).
14. Samuel G. Rodriques et al. Slide-seq: A scalable technology for measuring genome-wide expression at high spatial resolution. *Science* **363**, 1463-1467 (2019).
15. Ståhl, P. L. et al. Visualization and analysis of gene expression in tissue sections by spatial transcriptomics. *Science* **353**, 78–82 (2016).
16. Biermann, J. et al. Dissecting the treatment-naive ecosystem of human melanoma brain metastasis. *Cell* **185**, 2591-2608.e30 (2022).
17. Ji, A. L. et al. Multimodal Analysis of Composition and Spatial Architecture in Human Squamous Cell Carcinoma. *Cell* **182**, 497-514.e22 (2020).
18. Ma, Q. & Xu, D. Deep learning shapes single-cell data analysis. *Nat. Rev. Mol. Cell Biol.* **23**, 303–304 (2022).
19. Kinker, G.S. et al. Pan-cancer single-cell RNA-seq identifies recurring programs of cellular heterogeneity. *Nat. Genet.* **52**, 1208–1218 (2020).
20. Su, J., Vargas, D. V. & Kouichi, S. One pixel attack for fooling deep neural networks. *IEEE Trans. Evol. Comput.* **23**, 828–841 (2019).

21. Wang, J. et al. Generalizing to Unseen Domains: A Survey on Domain Generalization. *IEEE Trans. Evol. Comput.* **35**, 8052-8072 (2023).
22. Xu, J., Xiao, L. & Lopez, A. M. Self-supervised Domain Adaptation for Computer Vision Tasks. *IEEE Access* **7**, 156694–156706 (2019).
23. Venkateswara, H., Eusebio, J., Chakraborty, S. & Panchanathan, S. Deep Hashing Network for Unsupervised Domain Adaptation. in *the IEEE conference on computer vision and pattern recognition* (CVPR, 2017)
24. Oelen, R. et al. Single-cell RNA-sequencing of peripheral blood mononuclear cells reveals widespread, context-specific gene expression regulation upon pathogenic exposure. *Nat Commun.* **13**, 3267 (2022).
25. Tian, L. et al. Benchmarking single cell RNA-sequencing analysis pipelines using mixture control experiments. *Nat. Methods* **16**, 479–487 (2019).
26. Riemondy, K. A. et al. Neoplastic and immune single-cell transcriptomics define subgroup-specific intra-tumoral heterogeneity of childhood medulloblastoma. *Neuro-Oncol.* **24**, 273–286 (2022).
27. Bondoc, A. et al. Identification of distinct tumor cell populations and key genetic mechanisms through single cell sequencing in hepatoblastoma. *Commun. Biol.* **4**, 1049 (2021).
28. Szczerba, B. M. et al. Neutrophils escort circulating tumour cells to enable cell cycle progression. *Nature* **566**, 553–557 (2019).
29. Donato, C. et al. Hypoxia Triggers the Intravasation of Clustered Circulating Tumor Cells. *Cell Rep.* **32**, 108105 (2020).
30. Qian, J. et al. A pan-cancer blueprint of the heterogeneous tumor microenvironment revealed by single-cell profiling. *Cell Res.* **30**, 745–762 (2020).
31. Eberhardt, C. S. et al. Functional HPV-specific PD-1+ stem-like CD8 T cells in head and neck cancer. *Nature* **597**, 279–284 (2021).
32. Comprehensive analysis of spatial architecture in primary liver cancer. Sci. Adv. (2021).
33. Qi, J. et al. Single-cell and spatial analysis reveal interaction of FAP+ fibroblasts and SPP1+ macrophages in colorectal cancer. *Nat. Commun.* **13**, 1742 (2022).
34. Meylan, M. et al. Tertiary lymphoid structures generate and propagate anti-tumor antibody-producing plasma cells in renal cell cancer. *Immunity* **55**, 527-541.e5 (2022).
35. Huang, Z. et al.. Self-Challenging Improves Cross-Domain Generalization. in *Computer Vision–ECCV 2020* (*Computer Vision–ECCV*, 2020).
36. Ganin, Y. & Lempitsky, V. Unsupervised Domain Adaptation by Backpropagation. *in International conference on machine learning* 1180-1189 (ICML, 2015).
37. Ganin, Y. et al. Domain-Adversarial Training of Neural Networks. *J. Mach. Learn. Res.* **17**, 1-35 (2016).
38. Gretton, A., Borgwardt, K. M., Rasch, M. J. & Sch, B. A kernel two-sample test. *J. Mach. Learn. Res.* **13**, 723-773 (2012).
39. Sun, B. & Saenko, K. Deep CORAL: Correlation Alignment for Deep Domain Adaptation. in *Computer Vision–ECCV 2016* (*Computer Vision–ECCV*, 2016).
40. Zhang, H., Cisse, M., Dauphin, Y. N. & Lopez-Paz, D. mixup: Beyond Empirical Risk Minimization. in *International Conference on Learning Representations* (ICLR, 2018).
41. Sagawa, S., Koh, P. W., Hashimoto, T. B. & Liang, P. Distributionally Robust Neural Networks for Group Shifts: On the Importance of Regularization for Worst-Case Generalization. in *International*

Conference on Learning Representations (ICLR,2019).
42. Parascandolo, G., Neitz, A., Orvieto, A., Gresele, L. & Schölkopf, B. Learning explanations that are hard to vary. in *International Conference on Learning Representations* (ICLR, 2021).
43. Lu, W., Wang, J., Li, H., Chen, Y. & Xie, X. Domain-invariant Feature Exploration for Domain General- ization. Preprint at https://arxiv.org/abs/2207.12020 (2022).
44. Gunsagar S. Gulati et al. Single-cell transcriptional diversity is a hallmark of developmental potential. *Science* **367**,405-411 (2020).
45. Wang, L. et al. The Phenotypes of Proliferating Glioblastoma Cells Reside on a Single Axis of Variation. *Cancer Discov.* **9**, 1708–1719 (2019).
46. Kim, N. et al. Single-cell RNA sequencing demonstrates the molecular and cellular reprogramming of metastatic lung adenocarcinoma. *Nat. Commun.* **11**, 2285 (2020).
