# Supplementary material for: Domain generalization enables general cancer cell annotation in single-cell and spatial transcriptomics
Source: Nat Commun. 2024 Mar 2;15:1929. doi: 10.1038/s41467-024-46413-6 (PMC10908802; doi:10.1038/s41467-024-46413-6)
Supplement: Supplementary file 3 — Description of Additional Supplementary Files [file 41467_2024_46413_MOESM3_ESM.pdf]

<u>Description of Additional Supplementary Files</u>

Supplementary Data 1: Detailed precision and recall for AUPRC curves in Supplementary Figure 5.
