# Supplementary material for: Domain generalization enables general cancer cell annotation in single-cell and spatial transcriptomics
Source: Nat Commun. 2024 Mar 2;15:1929. doi: 10.1038/s41467-024-46413-6 (PMC10908802; doi:10.1038/s41467-024-46413-6)
Supplement: Supplementary file 5 — Reporting Summary [file 41467_2024_46413_MOESM5_ESM.pdf]

nature portfolio

Corresponding author(s): Jia Song

Last updated by author(s): Feb 4, 2024

Reporting Summary

Nature Portfolio wishes to improve the reproducibility of the work that we publish. This form provides structure for consistency and transparency in reporting. For further information on Nature Portfolio policies, see our Editorial Policies and the Editorial Policy Checklist.

Statistics

For all statistical analyses, confirm that the following items are present in the figure legend, table legend, main text, or Methods section.

n/a	Confirmed	
☐	☒	The exact sample size (*n*) for each experimental group/condition, given as a discrete number and unit of measurement
☐	☒	A statement on whether measurements were taken from distinct samples or whether the same sample was measured repeatedly
☐	☒	The statistical test(s) used AND whether they are one- or two-sided<br>*Only common tests should be described solely by name; describe more complex techniques in the Methods section.*
☒	☐	A description of all covariates tested
☐	☒	A description of any assumptions or corrections, such as tests of normality and adjustment for multiple comparisons
☐	☒	A full description of the statistical parameters including central tendency (e.g. means) or other basic estimates (e.g. regression coefficient) AND variation (e.g. standard deviation) or associated estimates of uncertainty (e.g. confidence intervals)
☐	☒	For null hypothesis testing, the test statistic (e.g. *F*, *t*, *r*) with confidence intervals, effect sizes, degrees of freedom and *P* value noted<br>*Give P values as exact values whenever suitable.*
☒	☐	For Bayesian analysis, information on the choice of priors and Markov chain Monte Carlo settings
☐	☒	For hierarchical and complex designs, identification of the appropriate level for tests and full reporting of outcomes
☐	☒	Estimates of effect sizes (e.g. Cohen's *d*, Pearson's *r*), indicating how they were calculated

Our web collection on statistics for biologists contains articles on many of the points above.

Software and code

Policy information about availability of computer code

Data collection	No software was used for data collection. The collected data was directly downloaded.
Data analysis	The Cancer-Finder was used for training and predicting. Model training and result analysis were performed using custom Python(3.9.16) scripts. Deep learning models involved in this study were performed by several standard libraries: Python(3.9.16), torch(1.13.1), numpy(1.23.0), pandas(1.5.2), scanpy(1.9.3), torchvision(0.14.1), tables(3.8.0). The code of Cancer-Finder is available under the MIT license at https://github.com/Patchouli-M/SequencingCancerFinder.The ccRCC spatial transcriptomes datasets with the Space Ranger(1.3.0) output form were processed by the Seurat(4.3.0) pipeline under R environment(4.2.2). Gene program extraction was applied via cNMF(1.4). TPM value of the ccRCC TCGA dataset were acquired from the UCSC xena database. ssGSEA scoring was achieved by GSVA(1.46.0) algorithm. Survival analysis were performed by survival(3.5-5) and survminer(0.4.9) package.

For manuscripts utilizing custom algorithms or software that are central to the research but not yet described in published literature, software must be made available to editors and reviewers. We strongly encourage code deposition in a community repository (e.g. GitHub). See the Nature Portfolio guidelines for submitting code & software for further information.

Data

Policy information about availability of data

All manuscripts must include a data availability statement. This statement should provide the following information, where applicable:

- Accession codes, unique identifiers, or web links for publicly available datasets
- A description of any restrictions on data availability
- For clinical datasets or third party data, please ensure that the statement adheres to our policy

All data used in this study were previously published and publicly available. The training and validation data used in this study are available in the TISCH database (http://tisch1.comp-genomics.org/). Most external validation single-cell data used in this study are available in the Gene Expression Omnibus (GEO) under accession codes: cell line data, GSM3618014 [https://www.ncbi.nlm.nih.gov/geo/query/acc.cgi?acc=GSM3618014]; medulloblastoma data, GSE155446 [https://www.ncbi.nlm.nih.gov/geo/query/acc.cgi?acc=GSE155446]; hepatoblastoma data, GSE180665 [https://www.ncbi.nlm.nih.gov/geo/query/acc.cgi?acc=GSE180665]; head and neck cancer data, GSE180268 [https://www.ncbi.nlm.nih.gov/geo/query/acc.cgi?acc=GSE180268]; breast cancer data, GSE148673 [https://www.ncbi.nlm.nih.gov/geo/query/acc.cgi?acc=GSE148673]; circulating tumor cells data, GSE109761 [https://www.ncbi.nlm.nih.gov/geo/query/acc.cgi?acc=GSE109761]. The remaining single-cell data for external validation, derived from a prior pancreatic cancer study, are accessible on the website (https://lambrechtslab.sites.vib.be/en/pan-cancer-blueprint-tumour-microenvironment-0). Please note that accessing this pancreatic cancer data requires registration. Peripheral blood mononuclear cell data from healthy donors used in this study are available on the website of 10x Genomics (https://www.10xgenomics.com/resources/datasets/10-k-peripheral-blood-mononuclear-cells-pbm-cs-from-a-healthy-donor-single-indexed-3-1-standard-4-0-0). Please note that accessing these data from 10x Genomics website requires registration. For spatial transcriptome data, colorectal cancer data used in this study are available in the Genome Sequence Archive (GSA) under accession code HRA000979 [https://ngdc.cncb.ac.cn/gsa-human/browse/HRA000979], hepatocellular carcinoma and intrahepatic cholangiocarcinoma data used in this study are available in the GSA under accession code HRA000437 [https://ngdc.cncb.ac.cn/gsa-human/browse/HRA000437]. Please note that access to these GSA data is restricted, and requests for access can be made through the GSA access committee. 10x Visium spatial transcriptome data for breast and ovarian cancer used in this study are available on the 10x Genomics website (BRCA1, https://www.10xgenomics.com/resources/datasets/human-breast-cancer-block-a-section-1-1-standard-1-1-0; BRCA2: https://www.10xgenomics.com/resources/datasets/invasive-ductal-carcinoma-stained-with-fluorescent-cd-3-antibody-1-standard-1-2-0; and ovarian cancer data, https://www.10xgenomics.com/resources/datasets/human-ovarian-cancer-whole-transcriptome-analysis-stains-dapi-anti-pan-ck-anti-cd-45-1-standard-1-2-0). Please note that accessing these data from 10x Genomics website requires registration. 10x Visium ST data from renal cell carcinoma (for training and identifying the gene signature) used in this study are available in the GEO under the accession code GSE175540 [https://www.ncbi.nlm.nih.gov/geo/query/acc.cgi?acc=GSE175540]. Renal cell carcinoma data with tumor-normal interface used in this study are available on the website https://data.mendeley.com/datasets/g67bkbnhhg/1. Data from other ST technologies (Slide-seq, legacy ST, and MERFISH) used in this study are available in the GEO under accession code GSE200278 [https://www.ncbi.nlm.nih.gov/geo/query/acc.cgi?acc=GSE200278], GSE144239 [https://www.ncbi.nlm.nih.gov/geo/query/acc.cgi?acc=GSE144239] and the website of MERSCOPE (https://info.vizgen.com/ffpe-showcase), respectively. Please note that accessing these MERSCOPE data requires registration. Source data are provided with this paper.

Research involving human participants, their data, or biological material

Policy information about studies with human participants or human data. See also policy information about sex, gender (identity/presentation), and sexual orientation and race, ethnicity and racism.

Reporting on sex and gender	n/a
Reporting on race, ethnicity, or other socially relevant groupings	n/a
Population characteristics	n/a
Recruitment	n/a
Ethics oversight	n/a

Note that full information on the approval of the study protocol must also be provided in the manuscript.

Field-specific reporting

Please select the one below that is the best fit for your research. If you are not sure, read the appropriate sections before making your selection.

☒ Life sciences ☐ Behavioural & social sciences ☐ Ecological, evolutionary & environmental sciences

For a reference copy of the document with all sections, see nature.com/documents/nr-reporting-summary-flat.pdf

Life sciences study design

All studies must disclose on these points even when the disclosure is negative.

Sample size	The sample size of each experiment for this study is showed in the manuscript, Supplementary information File and the corresponding figure/table legends. We did not perform sample size calculations. For training and testing, we collected as many cells/spots as possible based on

the available data to make the results more high-performance and generalized. For the number of repetitions for each experiment, we referred to the design in the most published studies.

Data exclusions	For training, three of tissue datasets (bladder, kidney, lymph nodes) were excluded because of the absence of cancer cells. Before the analysis of ccRCC, according to the original study, three of them were technical duplicates and two of them failed to pass the quality control and were thus excluded from subsequent analysis. The exclusion criteria were predefined.
Replication	The number of repetitions for each experiment was described in the legends of figures or tables in the manuscript and supplementary information files. And all attempts for replication were displayed.
Randomization	There were no study groups, randomization was therefore not relevant.
Blinding	There were no study groups, blinding was therefore not relevant.

Reporting for specific materials, systems and methods

We require information from authors about some types of materials, experimental systems and methods used in many studies. Here, indicate whether each material, system or method listed is relevant to your study. If you are not sure if a list item applies to your research, read the appropriate section before selecting a response.

Materials & experimental systems

n/a	Involved in the study	
☒	☐	Antibodies
☒	☐	Eukaryotic cell lines
☒	☐	Palaeontology and archaeology
☒	☐	Animals and other organisms
☒	☐	Clinical data
☒	☐	Dual use research of concern
☒	☐	Plants

Methods

n/a	Involved in the study	
☒	☐	ChIP-seq
☒	☐	Flow cytometry
☒	☐	MRI-based neuroimaging
